# International trends in radial artery usage for coronary artery bypass grafting

**DOI:** 10.1093/ehjopen/oeaf086

**Published:** 2025-06-28

**Authors:** Arnaldo Dimagli, Kevin R An, Sigrid Sandner, Polina Mantaj, Aina Hirofuji, C David Mazer, Bjorn Redfors, Feng Qiu, Stephen Fremes, Harindra C Wijeysundera, Thomas Schwann, Robert Habib, Mario Gaudino

**Affiliations:** Department of Cardiothoracic Surgery, Weill Cornell Medicine, 525 E 68th St, New York, NY 10065, USA; Department of Surgery, Columbia University Irving Medical Center, 177th Street Ft Washington Ave, New York, NY 10032, USA; Department of Cardiothoracic Surgery, Weill Cornell Medicine, 525 E 68th St, New York, NY 10065, USA; Division of Cardiac Surgery, Department of Surgery, University of Toronto, Stewart building, 149 College St, Toronto, Canada ON M5T 1P5; Department of Cardiothoracic Surgery, Weill Cornell Medicine, 525 E 68th St, New York, NY 10065, USA; Department of Cardiac Surgery, Medical University of Vienna, Währinger Gürtel 18-20, 1090 Vienna, Austria; Department of Cardiothoracic Surgery, Weill Cornell Medicine, 525 E 68th St, New York, NY 10065, USA; Department of Cardiothoracic Surgery, Weill Cornell Medicine, 525 E 68th St, New York, NY 10065, USA; Department of Anesthesia, St. Michael’s Hospital, University of Toronto, 30 Bond St, Toronto, Canada ON M5B 1W8; Department of Cardiothoracic Surgery, Weill Cornell Medicine, 525 E 68th St, New York, NY 10065, USA; Department of Cardiology, Sahlgrenska University Hospital, Blå stråket 5, 413 45 Göteborg, Sweden; Institute for Clinical Evaluative Sciences, 2075 Bayview Ave V Wing, Toronto, Canada ON M4N 3M5; Division of Cardiac Surgery, Department of Surgery, University of Toronto, Stewart building, 149 College St, Toronto, Canada ON M5T 1P5; Schulich Heart Program, Sunnybrook Health Sciences Centre, University of Toronto, Hospital Road, Toronto, Canada ON M4N 3M5; Department of Cardiovascular Surgery, Corewell East William Beaumont University Hospital, Royal Oak, 3601 13 Mile Rd, Royal Oak, MI 48073, USA; STS Research and Analytic Center, Chicago, 633 N Saint Clair St, Suite 2100, Chicago, IL 60611, USA; Department of Cardiothoracic Surgery, Weill Cornell Medicine, 525 E 68th St, New York, NY 10065, USA

**Keywords:** CABG, Radial artery, National database

## Abstract

**Aims:**

The study aimed to investigate international trends in the adoption of the radial artery (RA) as a conduit for coronary artery bypass grafting across different national and regional registries.

**Methods and results:**

Data were extracted from four databases: the UK cardiac surgery database, the Ontario provincial administrative database, the Austrian national adult cardiac surgery database, and the Society of Thoracic Surgeons Adult Cardiac Surgery Database (STS ACSD). Radial artery use rates were 4.3% in the UK, 23.3% in Ontario, 4.8% in Austria, and 6.4% in the STS ACSD. Significant uptrends in RA use were observed in Ontario (*P* = 0.001), Austria (*P* = 0.004), and the STS ACSD (*P* = 0.02), while a downtrend was noted in the UK (*P* = 0.015). Endoscopic RA harvesting was increasingly adopted, particularly in Ontario and the STS ACSD.

**Conclusion:**

Global adoption of RA remains variable and generally low with a general uptrend and higher adoption of endoscopic harvesting.

The current European and American guidelines for coronary artery revascularization recommend the use of radial artery (RA) over the saphenous vein during coronary artery bypass grafting (CABG) to graft the second most important, significantly-stenosed, non-left anterior descending coronary target.^1,2^ This recommendation is based on current evidence showing superior long-term RA patency and lower rates of adverse cardiac events.^3–6^ The implementation of guidelines in surgical practice has often been variable, and adoption rates may vary depending on regional practices, training, and healthcare infrastructures. Robust data on global adoption of the RA for CABG are lacking; the aim of this study is to review and document international trends in the use of the RA for CABG.

Data for this analysis were derived from four databases: the national UK cardiac surgery database (2008–2019), the Ontario provincial administrative database (2008–2023), the Austrian national adult cardiac surgery database (2009–2022), and the Society of Thoracic Surgeons Adult Cardiac Surgery database (STS ACSD; 2008–2023). Data were extracted and aggregated from the individual registries for UK (*n* = 371 092), Ontario (*n* = 104 413), and Austria (*n* = 35 892) and extracted from publicly available sources for the STS ACSD (*n* = 2 536 544). As the study included retrospective interrogation of databases and/or publicly available data, no institutional board approval was requested. In each database, the rate of RA use was calculated as a percentage of the annual number of isolated CABG surgeries performed. Trends of RA use over time were evaluated using Mann–Kendall trend test, with statistical significance set at *P* < 0.05 with no multiplicity adjustment. When available, data about RA harvesting technique were also reported.

The overall average use of RA was 4.3% in the UK, 23.3% in Ontario, 4.8% in Austria, and 6.4% in the STS ACSD.

Temporal trends are shown in *Figure 1*. In the UK database, the use of the RA showed an initial decline in use from 7.8% in 2008 to 3.2% in 2014 followed by a slow but steady uptrend to 4% in 2019; overall, there was a significant downtrend in the use of RA in the UK (*P*_trend_ = 0.015). In Ontario, RA adoption showed a slow uptrend (*P*_trend_ = 0.001) with a use rate of 25.2% in 2008 to 29.2% in 2023. The Austrian data showed a significant uptrend (*P*_trend_ = 0.004) from 2009 (2.9%) to 2022 (6.4%). Similarly, the STS ACSD data showed an initial RA use of 6.2% in 2008 that peaked to 10.4% in 2023 with significant uptrend (*P*_trend_ = 0.02).

**Figure 1:**
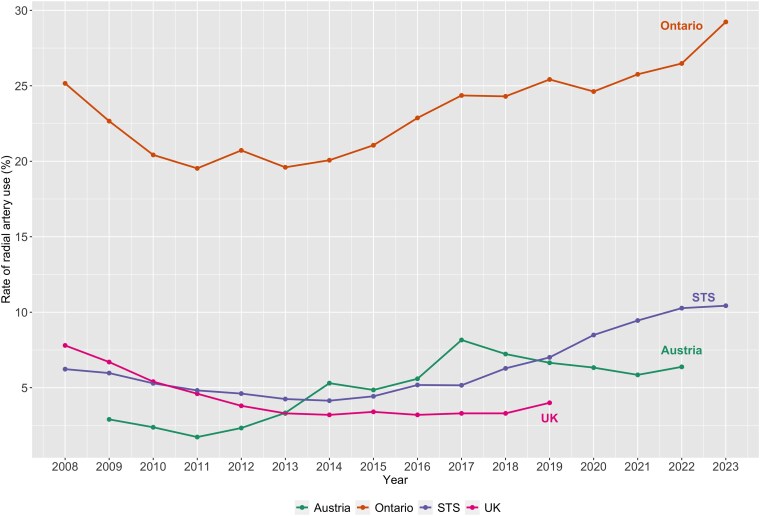
Yearly rates of radial artery use in four national/regional databases. STS, Society of Thoracic Surgeons; UK, United Kingdom.

In the Ontario database, endoscopic harvesting of RA was absent in 2008 but peaked at 14% in 2020. Similarly, rates of endoscopic RA were higher in the latest years (58.2%) in the STS ACSD.^7–9^

This study shows that the adoption of RA for CABG is widely variable among different national/regional registries, with a general uptrend in RA use and the highest rate observed in Canada. Specifically, the rate in Ontario exceeds 20%, a remarkable outlier that may reflect region-specific clinical guidelines, procedural expertise concentration, or incentivized institutional practices. Interestingly, in three of the databases, there was an uptrend starting after 2018, year of publication of the European guidelines on myocardial revascularization which for first time recommended the use of the RA as class I.^2^ However, the current adoption rates seem rather low considering the Level I recommendation by the current guidelines.^1,2^ In the RAPCO trial, there was a lower rate of major cardiovascular events for the RA when compared with either the right internal thoracic artery (−26%) or the saphenous vein (−29%).^6^ In a meta-analysis of four large CABG trials, the RA was associated with lower all-cause mortality and major adverse cardiovascular events when compared to the saphenous vein and the right internal thoracic artery.^3^

Despite the current data in support of the use of the RA, there could be hesitation from surgeons due to a perceived higher peri-operative risk and technical complexity in RA use. It is important to note that the RA is easily accessible, can be harvested simultaneously with other arterial grafts optimizing surgery times, and has optimal calibre and length lending itself to multiple practical configurations.^10^ In comparison to the right internal thoracic artery, the RA offers the added benefit of simpler technical handling without increasing the risk of sternal wound complications.^10^ Moreover, endoscopic harvesting using minimally invasive technique performed through small incisions under videoscopic guidance could be associated with lower postoperative pain and increased patient satisfaction^11^ without impacting long-term survival and graft patency.^12,13^

These findings are limited by the absence of patient-level data, potential regional variability in practice and reporting, different temporal frames (UK data available only until 2019), and possible impact of the COVID-19 pandemic on surgical trends.

In conclusion, current data suggest that RA adoption is variable in different registries, but a general uptrend and higher adoption of endoscopic harvesting were noted. Given the evidence supporting the safety and efficacy of RA, initiatives to promote its broader adoption are justified and should be prioritized in future practice guidelines and quality improvement efforts.

## Data Availability

The data underlying this article are available in the article.
